# Use of Optical Imaging Technology in the Validation of a New, Rapid, Cost-Effective Drug Screen as Part of a Tiered *In Vivo* Screening Paradigm for Development of Drugs To Treat Cutaneous Leishmaniasis

**DOI:** 10.1128/AAC.02048-16

**Published:** 2017-03-24

**Authors:** Diana Caridha, Sandi Parriot, Thomas H. Hudson, Thierry Lang, Franklyn Ngundam, Susan Leed, Jenell Sena, Michael Harris, Michael O'Neil, Richard Sciotti, Lisa Read, Herve Lecoeur, Mark Hickman, Max Grogl

**Affiliations:** a Walter Reed Army Institute of Research, Experimental Therapeutics Branch of the Military Malaria Research Program, Silver Spring, Maryland, USA; b Institut Pasteur, Unité d'Immunophysiologie et Parasitisme Intracellulaire, Département de Parasitologie et Mycologie, Paris, France

**Keywords:** cutaneous leishmaniasis, Golden Syrian hamster, BALB/c mouse, *in vivo* imaging system, Leishmania lesion cure, Leishmania lesion suppression, antileishmanial drugs, bioluminescence signal, *in vivo* drug screen, mouse models, transgenic Leishmania major

## Abstract

In any drug discovery and development effort, a reduction in the time of the lead optimization cycle is critical to decrease the time to license and reduce costs. In addition, ethical guidelines call for the more ethical use of animals to minimize the number of animals used and decrease their suffering. Therefore, any effort to develop drugs to treat cutaneous leishmaniasis requires multiple tiers of *in vivo* testing that start with higher-throughput efficacy assessments and progress to lower-throughput models with the most clinical relevance. Here, we describe the validation of a high-throughput, first-tier, noninvasive model of lesion suppression that uses an *in vivo* optical imaging technology for the initial screening of compounds. A strong correlation between luciferase activity and the parasite load at up to 18 days postinfection was found. This correlation allows the direct assessment of the effects of drug treatment on parasite burden. We demonstrate that there is a strong correlation between drug efficacy measured on day 18 postinfection and the suppression of lesion size by day 60 postinfection, which allows us to reach an accurate conclusion on drug efficacy in only 18 days. Compounds demonstrating a significant reduction in the bioluminescence signal compared to that in control animals can be tested in lower-throughput, more definitive tests of lesion cure in BALB/c mice and Golden Syrian hamsters (GSH) using Old World and New World parasites.

## INTRODUCTION

The World Health Organization (WHO) estimates an incidence of 2 million new cases of leishmaniasis per year (0.5 million cases of visceral leishmaniasis and 1.5 million cases of cutaneous leishmaniasis [CL]), with more than 350 million people being at risk of infection (WHO Technical Report Series 949). There is a compelling need for oral treatment of cases of CL that do not rapidly self-cure, present numerous lesions (typically, more than four), and have the potential to cause mucocutaneous leishmaniasis (MCL) (1–4).

Recent reviews emphasize the need for *in vivo* animal models of CL with clinical similarities to the human form that ensure a positive correlation between the *in vitro* potency of an antileishmanial drug and its *in vivo* efficacy before human clinical trials begin (5). Models of *in vivo* lesion (dorsal and footpad) cure using the BALB/c mouse and Golden Syrian hamster (GSH) with Leishmania spp. as a source of CL have been widely used to test the efficacies of antileishmanial drugs (4–10). Leishmania major infection triggers a strong Th2 response in BALB/c mice, which lack an early NK cell response and a Th1 type of response. As a result, rapid lesion growth and severe cutaneous disease are observed (5). Even though the BALB/c mouse-L. major model does not accurately reproduce the biological responses that occur in humans, this model is rigorous, convenient, and reproducible (5). Furthermore, BALB/c mice develop lesions that are similar to those in patients with CL (5). GSH have been described by some authors to be one of the best models for CL because of their susceptibility to infection by different Leishmania spp. and the fact that the clinical evolution of lesions observed in GSH is similar to that observed in humans (6, 8, 11). As in humans, the lesions in GSH vary in size depending on the immune status of each individual, and therefore, a reduction in lesion size or spontaneous lesion healing is often observed (6, 8, 11).

However, while a model that tests leishmania lesion cure is the most definitive *in vivo* model possible, use of this model is costly and time-consuming and the model requires a long incubation period before observations can be made and is not suitable for first-in-animal testing. Moreover, the measurement of lesion size, the principal endpoint in a lesion cure assay, has some drawbacks, and various publications have suggested that the parasite load may well be a better indication of the degree of infection (4). Available techniques utilized for parasite load measurements, such as analysis of biopsy specimens and PCRs, are invasive and time-consuming and do not allow for longitudinal studies (4, 5). Therefore, in the search for potential antileishmanial drugs, it is very important to be able to quantify the Leishmania parasite load in a live host (5). Several probes, such as the green fluorescent protein (GFP), enhanced green fluorescent protein (EGFP), mCherry red fluorescent protein, and near-infrared fluorescent proteins, as well as the firefly luciferase (LUC) reporter gene, have been stably integrated into the parasite genome and have been widely used to monitor the *in vivo* intracellular proliferation of Leishmania parasites (12–24). Taheri et al. have reported utilizing two reporter proteins, EGFP and LUC, to quantify the parasite load and increase the *in vivo* experimental sensitivity (25). Published work has shown a strong correlation between the parasite load and luciferase activity or fluorescence emission, which makes the use of transgenic Leishmania parasites a useful tool to monitor disease progression and the efficacy of antileishmanial drugs in animal models (14, 18, 20, 22).

In 2013, we published a review article defining a drug discovery algorithm and strategy for drugs that may be used to treat CL (28). In this publication, we present a validation of the *in vivo* screens that we introduced in our review article. In brief, this gated-tier testing paradigm progresses compounds with *in vitro* potency and good chemical characteristics (low or no *in vitro* toxicity, favorable stability in liver microsomes, acceptable permeability, etc.) into a first-tier model of the efficacy of lesion suppression in highly susceptible immunodeficient BALB/c mice, followed by a second-tier lesion cure model in BALB/c mice and immunocompetent GSH. The use of two different rodent models with various degrees of immune response to Leishmania infection provides a strong set of tools to examine the efficacy of antileishmanial drugs. The first-tier, higher-throughput model of *in vivo* suppression in BALB/c mice infected with transgenic luciferase-expressing L. major parasites utilizes longitudinal *in vivo* imaging as an endpoint for measuring drug efficacy. In our studies, we utilized the transgenic luciferase-expressing L. major strain NIH173 (MHOM/IR/−/173), which was a gift from Genevieve Milon, Department of Parasitology and Mycology, Institut Pasteur, Paris, France, and was successfully used in prior published *in vivo* studies (14, 18). This new model of lesion suppression with real-time screening of drug activity using *in vivo* imaging in BALB/c mice is noninvasive, rapid, accurate, sensitive, and cost-effective, reduces the hit-to-lead cycle, and facilitates the selection of new candidate compounds that may advance to preclinical and early clinical studies. Moreover, this technique allows us to evaluate disease progression, parasite load, and drug efficacy before the clinical signs of advanced disease become present. Lead compounds are then tested in second-tier, more traditional models of lesion cure with BALB/c mouse and GSH that we have also validated. The tiered testing approach described here minimizes the numbers of animals used per test and maximizes the number of promising antileishmanial compounds to be tested.

## RESULTS

### *In vitro* characterization of the luciferase-expressing L. major parasites.

As shown in Fig. S1 in the supplemental material, there is a very strong *in vitro* correlation between the number of luciferase-expressing L. major parasites and luciferase activity (*R*^2^ = 0.99). The *in vitro* limit of detection for the luciferase-expressing L. major strain was determined to be ≥5,000 parasites (data not shown). The *in vitro* potencies (50% inhibitory concentrations [IC_50_s]) of some known antileishmanial compounds, such as amphotericin B, paromomycin, miltefosine, pentamidine, sodium stibogluconate (Pentostam), ketoconazole, and sitamaquine, against intracellular amastigotes were previously determined and are described elsewhere (26). The *in vitro* potencies of amphotericin B liposome for injection (AmBisome), glucantime, fluconazole, ofloxacin, and posaconazole in the intracellular amastigote assay and the *in vitro* toxicities (IC_50_s) of all tested antileishmanial compounds were determined in this study. The results are shown in Table S1. Except for pentamidine, which demonstrated high levels of toxicity *in vitro*, the majority of known antileishmanial compounds demonstrated high levels of *in vitro* efficacy and low levels of *in vitro* toxicity against the RAW 264.7 murine macrophage cell line.

### Disease progression in BALB/c mice infected with WT (MHOM/SU/74/WR779) and transgenic (MHOM/IR/−/173) L. major parasites.

When BALB/c mice were infected at the base of the tail with 1 × 10^7^ wild-type (WT) (MHOM/SU/74/WR779) parasites, disease progression was similar to that in mice infected with luciferase-expressing L. major. In mice infected with either strain, papules started developing at 9 to 15 days postinfection and opened, causing small lesions at 16 to 25 days postinfection. According to the results of our studies, 7 to 9% of the luciferase-expressing L. major parasites used for infection at this stage were metacyclic and routinely yielded a 100% infection rate.

### Determination of luciferin kinetics for models of transgenic L. major infection in BALB/c mice and GSH.

**FIG 1:**
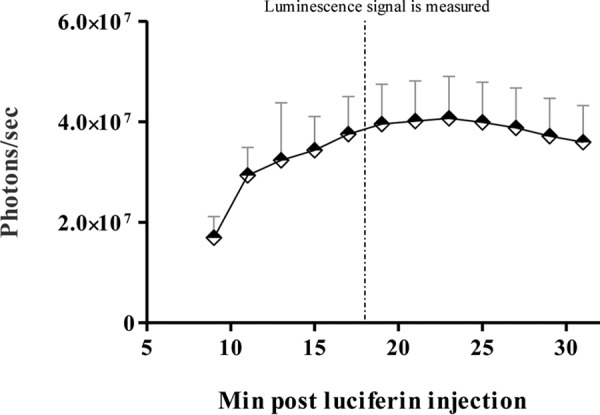
Luciferin kinetics in BALB/c mice. Luciferin was administered at 200 mg/kg i.p. to BALB/c mice on day 18 postinfection with 1 × 10^7^ stationary-phase L. major promastigotes. Bioluminescence measurements were taken every 2 min starting 8 min after luciferin administration. Bars represent the mean bioluminescence signal ± SEM for a total of 10 BALB/c mice for each experimental condition in two different studies.

A study was conducted to find the time after luciferin injection when the bioluminescence signal reached a plateau and bioluminescence measurements could best be determined (27). A group of five mice or three hamsters was monitored for this purpose on day 18 postinfection. Luciferin was administered to each BALB/c mouse and GSH intraperitoneally (i.p.) at 200 mg/kg of body weight. Animals were anesthetized and placed in the imaging chamber of the PerkinElmer IVIS Spectrum instrument, and consecutive bioluminescence readings were taken every 2 min for BALB/c mice and every 5 min for GSH. As shown in Fig. 1, in BALB/c mice the bioluminescence signal increased and peaked at approximately 20 min after luciferin injection. We decided to measure the bioluminescence signal for all subsequent experiments 18 min after luciferin injection, when the bioluminescence signal was shown to be high and the exposure time was short. In addition, the optimal settings for the IVIS machine for all drug testing studies was examined through iterative testing, and the most effective settings were found to be medium binning and automatic exposure. In GSH, the signal continued to increase over time and reached a plateau approximately 30 min after luciferin injection (data not shown), but for the same reasons mentioned above, we decided to measure the bioluminescence signal 20 min after luciferin injection with settings of medium binning and a 5-min exposure time.

### Assessment of the correlation between parasite load and bioluminescence signal in model of suppression of lesions formed by luciferase-expressing L. major in BALB/c mice.

**FIG 2:**
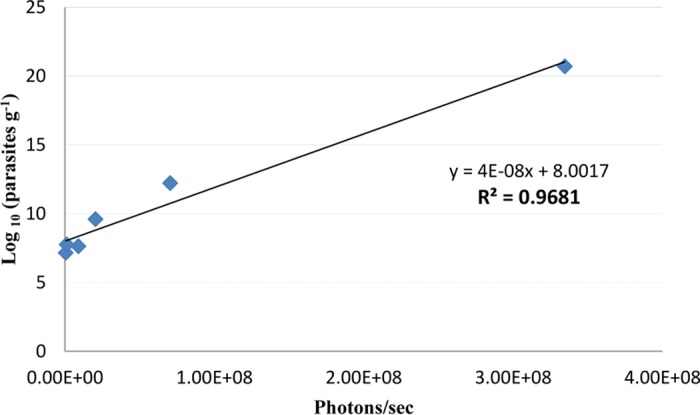
Correlation between log-transformed values of parasite load and bioluminescence signal up to day 18 postinfection. BALB/c mice were infected i.d. at the base of the tail with 1 × 10^7^ stationary-phase L. major parasites. Animals were then randomly chosen and euthanized on days 1, 3, 6, 9, 14, and 18 postinfection. For each data point, the bioluminescence signal (in number of photons per second) for each mouse was measured before sacrifice. Immediately after euthanasia, the infected tissue was excised from the dorsolumbar region using a punch biopsy and the parasite load was evaluated. Each point represents the mean parasite load and bioluminescence signal measured for 3 mice.

**FIG 3:**
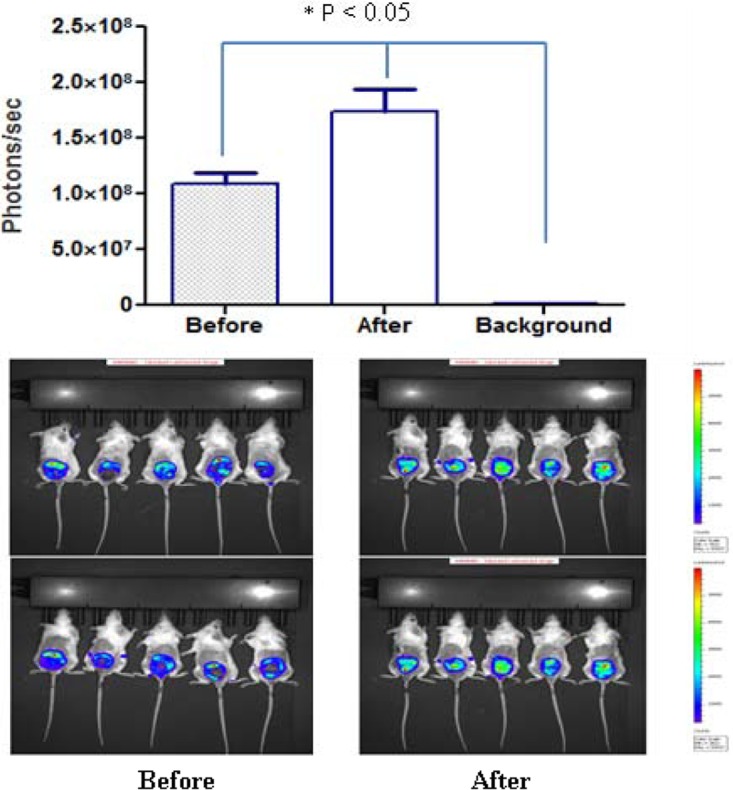
The presence of a scab attenuates the intensity of the bioluminescence signal at the lesion site. Two groups of five mice which were infected i.d. at the base of the tail with 1 × 10^7^ stationary-phase luciferase-expressing L. major parasites were imaged at 50 days postinfection. For each mouse, the total photon emission (in number of photons per second) from the L. major-loaded site was measured before and after scab removal. The photon intensity numbers in the brighter areas of the image (red or bright yellow) are greater than those in the areas with a dimmer green or blue color, where fewer photons were detected. Bars represent the mean ± SEM for a total of 10 mice for each experimental condition. The background bioluminescence signal was considered the signal emitted from intact skin located above the lesion site. A single-factor ANOVA (Dunnett's test) was used to determine whether differences in group means existed across the experiment. *, statistically significant differences (*P* < 0.05).

As shown in Fig. 2, there was a strong correlation between the bioluminescence signal and the parasite load (*R*^2^ = 0.96) up to 18 days postinfection. The bioluminescence signal emitted by the infection sites in BALB/c mice reached a plateau and/or began to diminish at approximately 35 days postinfection (data not shown). Given the fact that the luciferase gene and the construct used to integrate it into the Leishmania genome are quite stable, we believe that this characteristic of the bioluminescence signal is likely due to the signal attenuation associated with the appearance of a dark crust on lesions and not to an intrinsic decrease of the signal *in vivo* (5). Dark crusts started to appear on the surface of Leishmania lesions at approximately 18 to 25 days postinfection, and this crust could easily quench the bioluminescence signal and thus attenuate the bioluminescence measured. As shown in Fig. 3, when the dark crust that covered the lesion was removed, the bioluminescence signal increased significantly (*P* < 0.05). Moreover, the accumulation of necrotic tissue at the site of infection might further reduce the bioluminescence signal. For this reason, the bioluminescence signal data observed for each animal were collected and followed as a means for evaluating the presence (or lack thereof) of parasites at the infection site, but the bioluminescence signal was not used as an experimental endpoint in the lesion cure models.

### Benchmarking of lesion suppression activity of known antileishmanial compounds using the BALB/c mouse model of L. major lesion suppression.

The activities of several standard antileishmanial drugs were evaluated in the BALB/c mouse model of luciferase-expressing L. major lesion suppression (Table 1). The drugs used to benchmark this model included three formulations of amphotericin B (amphotericin B, amphotericin B lipid complex [ABLC], and AmBisome), sodium stibogluconate (Pentostam), miltefosine, posaconazole, fluconazole, ofloxacin, glucantime, sinefungin, paromomycin, and pentamidine. In order to maximize bioavailability, drugs were administered i.p., except for pentamidine, which was administered i.p. and intramuscularly (i.m.). The vehicle control (1× phosphate-buffered saline [PBS]) and the positive control (25 mg/kg AmBisome) were administered i.p. as well. The drugs were given for 10 consecutive days, except for paromomycin, sodium stibogluconate, sinefungin, and i.m. pentamidine, which were given for 7, 14, 8, and 3 days, respectively. Drug dosages were chosen on the basis of the human-equivalent dose (HED), and dosing calculations were based upon body weight measurements obtained on day 3 postinfection. Drugs were formulated as described in “Establishment of a first-tier Leishmania lesion suppression screening model using *in vivo* imaging in BALB/c mice infected with luciferase-expressing L. major parasites” in Materials and Methods.

**TABLE 1:** Bioluminescence signal and lesion suppression rates in BALB/c mice treated with known antileishmanial compounds^*a*^

Compound tested	Dose (mg/kg)	Bioluminescence signal suppression at 18 ± 1 days postinfection	Lesion suppression at 60 ± 2 days postinfection
Amphotericin B formulations			
Amphotericin B	2	−88.1	−70.7
Amphotericin B	4	−150.0	−33.0
Amphotericin B	6	36.5	66.7
Amphotericin B	8	67.3*	88.8*
Amphotericin B	16	99.1*	84.5*
ABLC	12.5	74.2*	60.9*
ABLC	25	93.1*	77.9*
ABLC	37.5	99.4*	96.5*
AmBisome	12.5	99.4*	80.7*
AmBisome	25	99.8*	99.1*
AmBisome	37.5	100*	100.0*
AmBisome	50	100*	100.0*
Other antileishmanial compounds			
Paromomycin	80	100*	100*
Sodium stibogluconate	400	89.7*	65.9
Pentamidine	50	68.9	63.1^&^
Sinefungin	10^*b*^	99.9*	Euthanized
Posaconazole	100	94.9*	96.5*
Ofloxacin	80	−52.8	−85.4
Miltefosine	17.5	18.1	−20.1
Fluconazole	160	−0.4	−10.6
Glucantime	100	−13.7	−29.8

a The lesion suppression activities of different doses of the three formulations of amphotericin B and other known antileishmanial compounds are shown. Five BALB/c mice were assigned to each treatment and vehicle-control group. Mice were infected i.d. with 1 × 10^7^ stationary-phase luciferase-expressing L. major parasites at the base of the tail on day 0. Drug treatments were initiated on day 3 postinfection. Drugs were given for 10 consecutive days, except for paromomycin, sodium stibogluconate, sinefungin, and i.m. pentamidine, which were given for 7, 14, 8, and 3 days, respectively. The mean bioluminescence signals at the infection site (in number of photons per second per mouse) and the mean lesion size (in square millimeters) were measured on days 18 ± 1 and 60 ± 2 postinfection, respectively, and were expressed as a percentage of the bioluminescence signal compared with that for the vehicle-control group or the mean percent lesion suppression compared with that for the vehicle-control group. A single-factor ANOVA (Dunnett's test) was used to determine whether differences in group means existed across the experiment. *, a statistically significant difference (*P* < 0.05); &, lesion size was last measured on day 33 postinfection.

b The bioluminescence signal was measured for the last time on day 11 postinfection, when all animals were euthanized due to drug toxicity (weight loss, ≥20%). As a result, only eight doses of sinefungin were given.

**FIG 4:**
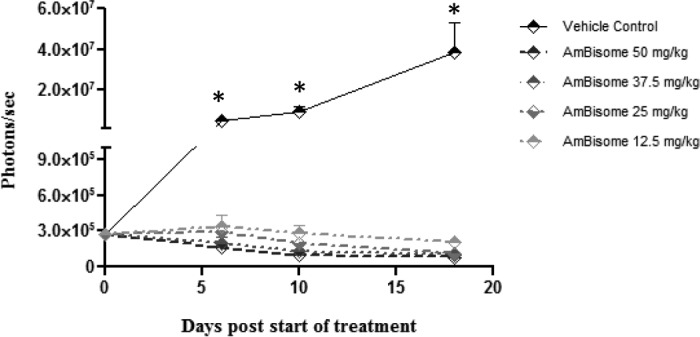
AmBisome reduces the parasite load at the infection site in a dose-dependent manner. The bioluminescence signal from BALB/c mice infected i.d. with 1 × 10^7^ stationary-phase L. major promastigotes and treated i.p. with 50, 37.5, 25, and 12.5 mg/kg AmBisome is plotted against the number of days after the start of treatment. All doses of AmBisome were given i.p. for 10 consecutive days. Bars represent the mean bioluminescence signal ± SEM for a total of five BALB/c mice. A single-factor ANOVA (Dunnett's test) was used to determine whether differences in group means existed across the experiment. *, statistically significant differences (*P* < 0.05).

**FIG 5:**
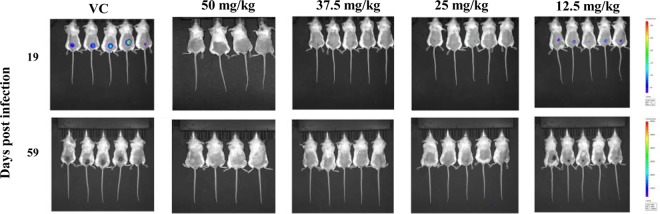
*In vivo* bioluminescence imaging on day 19 and the corresponding lesion sizes on day 59 postinfection in BALB/c mice treated with various doses of AmBisome in the lesion suppression model. Mice were infected i.d. with 1 × 10^7^ stationary-phase luciferase-expressing L. major parasites at the base of the tail on day 0. Drug treatments (12.5, 25, 37.5, and 50 mg/kg AmBisome) were initiated on day 3 postinfection. The bioluminescence signal was measured on day 19, and lesion sizes were followed and measured for the last time on day 59 postinfection. The bioluminescence signal represents the light intensity over the body surface area. Red and bright yellow represent the most intense signal, followed by green and then blue, which represents the weakest signal. The bioluminescence signal was detected at the infection site in all groups starting from day 0 postinfection and continued to be present in the vehicle-control (VC) group until the end of the study. The lack of a bioluminescence signal at the infection site suggested that the parasite load was nonexistent or below the limit of detection.

When the efficacies of known antileishmanial drugs were tested, the bioluminescence signal was measured every 3 to 4 days to evaluate the evolution of the parasite load. The bioluminescence signal was also measured for all treatment groups on day 18 postinfection (day 18 was chosen for measurement, as all drug treatments had ended by that time), was captured as the mean bioluminescence signal (in number of photons per second), and was expressed as a percentage of the mean bioluminescence signal observed in vehicle-control-treated animals (Table 1). The three formulations of amphotericin B were tested at several doses, and their efficacies in the BALB/c mouse model of the suppression of lesions caused by luciferase-expressing L. major are shown in Table 1. AmBisome was the most efficacious, followed by ABLC and amphotericin B. AmBisome was initially tested at 12.5, 25, 37.5, and 50 mg/kg, and as shown in Fig. 4, it was noted to yield efficacy results in a dose-dependent manner. The bioluminescence signal emitted from the transgenic L. major parasites fell below the limit of detection (LOD; which in our experimental model was ≤20,000 parasites; data not shown) before the 25-, 37.5-, and 50-mg/kg AmBisome treatments had ended. At 18 days postinfection, the bioluminescence signal correlating to the parasite load was still below the LOD in mice treated with 37.5 and 50 mg/kg AmBisome and was reduced by 99.8% and 99.4% in BALB/c mice treated with 25 mg/kg and 12.5 mg/kg AmBisome, respectively. At 60 days postinfection, no parasites were visible in the groups treated with 37.5 and 50 mg/kg AmBisome, and no lesions were observed in either group. When AmBisome was administered at 50 mg/kg, it caused weight loss in all mice in this treatment group, which was probably due to toxicity. At the end of the 10-day treatment, these animals had lost 6.1% of their overall body weight. One mouse was found dead in its cage; on the previous day this animal was found to have lost 18.2% of its body weight, which, according to our animal protocol, was just below the 20% body weight loss cutoff established for euthanasia. Two out of the five mice treated with 25 mg/kg AmBisome and five out of the five mice treated with 12.5 mg/kg Ambisome started developing small lesions at 48 and 33 days postinfection, respectively. The average lesion sizes measured at 60 days postinfection for the groups treated with 25 and 12.5 mg/kg AmBisome were reduced by 99.1% and 80.7%, respectively, compared to that for the vehicle-control group. In consecutive studies (not shown), two or three BALB/c mice treated with 25 mg/kg AmBisome had developed very small lesions at day 60 postinfection, but the majority of the animals showed no lesion formation. In some of these animals, a bioluminescence signal was detected toward the end of the 60-day waiting period even when lesions were not visible. On the basis of these findings, the 25-mg/kg AmBisome treatment was chosen to be the positive control for the BALB/c mouse model of lesion suppression. The bioluminescence signal on day 19 postinfection and the corresponding lesion sizes on day 59 postinfection for the AmBisome treatment are shown in Fig. 5.

When administered at 12.5, 25, and 37.5 mg/kg, ABLC significantly reduced both the parasite load measured at the infection site on day 18 postinfection and the mean lesion size measured on day 60 postinfection compared to the results for the vehicle-control group. Still, ABLC failed to reduce the bioluminescence signal below the LOD at any of the concentrations mentioned above. Amphotericin B was tested at 2, 4, 6, 8, and 16 mg/kg, but only the 8- and 16-mg/kg treatments were found to significantly reduce the parasite load and the mean lesion size measured on days 18 and 59 postinfection, respectively. Both ABLC and amphotericin B reduced the parasite load and the mean lesion size in a dose-dependent manner.

The *in vivo* efficacies of paromomycin, sinefungin, sodium stibogluconate, glucantime, miltefosine, posaconazole, fluconazole, ofloxacin, and pentamidine are also shown in Table 1. Assessment of paromomycin (80 mg/kg) was conducted over a treatment period of 7 days. The bioluminescence signal at the infection site fell below the LOD before the treatment ended and stayed undetectable up to 45 days postinfection. Furthermore, these animals did not develop lesions during the 2-month follow-up period. Sinefungin treatment (initially planned for 14 days at 10 mg/kg/day) reduced the parasite load by 99.9%; however, treatment was stopped after eight doses of the drug were given and all animals were euthanized due to significant weight loss (≥20%). Sodium stibogluconate was administered at 400 mg/kg for 14 consecutive days. By day 18 postinfection, the bioluminescence signal was reduced by 89.7% and the corresponding mean lesion size on day 60 postinfection was reduced by 65.9% (the difference was not statistically significant, maybe due to the high degree of variability of lesion sizes across the group) compared to the results for the vehicle-control group. Fluconazole (160 mg/kg), ofloxacin (80 mg/kg), glucantime (100 mg/kg), and miltefosine (17.5 kg) were also tested but did not show antileishmanial efficacy in the mouse model of lesion suppression. Posaconazole, which was administered at 100 mg/kg for a period of 10 days, reduced the parasite load and suppressed the lesion size by 94.9% and 96%, respectively, compared to the results for the vehicle-control group. Of all of the azole compounds tested, posaconazole, a newer broad-spectrum triazole, was the only compound that showed good *in vivo* efficacy against L. major parasites in this particular screen. Pentamidine failed to reduce the parasite load when it was administered at 5 and 10 mg/kg i.p. for 10 consecutive days (data not shown). In a follow-on study, 50 mg/kg pentamidine was administered three times i.m. on days 3, 5, and 7 postinfection. The mean bioluminescence signal and the mean lesion size measured on days 18 and 60 postinfection were reduced by 68.9% and 63.1%, respectively, compared to the results for the vehicle-control group.

### Suppression of the bioluminescence signal on the first day after the end of treatment correlates with suppression of lesion size in late-stage CL disease in BALB/c mice.

BALB/c mice that were treated with known antileishmanials (as described above) were kept for up to 60 ± 2 days postinfection, at which time the size of the lesion (if any lesion was present) was measured and the percent lesion size suppression compared to the lesion size for the animals in the vehicle-control group was calculated (Table 1). There was a strong correlation (*R*^2^ = 0.80) between suppression of the bioluminescence signal on the first day after the end of treatment and suppression of the lesion size in late-stage CL disease in BALB/c mice. These data support our hypothesis that a lesion suppression assay conducted with *in vivo* imaging technology is capable of predicting whether a CL lesion will actually develop and, if it does, what the approximate mean lesion size in the treated group will be.

### Assessment of efficacy of treatment with topical antileishmanial creams with *in vivo* imaging technology.

**FIG 6:**
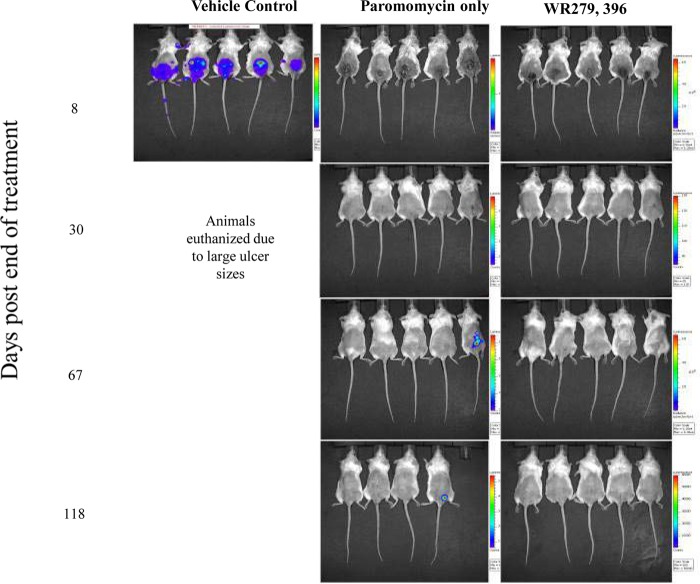
Assessment of effects of topical paromomycin and WR279396 on the bioluminescence signal (parasite load) from BALB/c mice infected with the transgenic L. major parasite. Mice were infected i.d. with 1 × 10^7^ L. major luciferase-expressing stationary-phase promastigotes at the base of the tail, and lesions were followed until the mean lesion size was approximately 50 mm^2^. Subsequently, they were assigned to three different study groups in which lesion sizes were not statistically significantly different. Vehicle-control, paromomycin, and WR279396 creams were applied BID for 10 consecutive days. The bioluminescence signal was followed as a means for evaluating the presence (or lack thereof) of parasites at the infection site. The bioluminescence signal represents light intensities over the body surface area, with red indicating the most intense signal and blue indicating the weakest signal. A lack of a bioluminescence signal at the infection site suggested that the parasite load was nonexistent or below the limit of detection. A single-factor ANOVA (Dunnett's test) was used to determine differences in mean lesion sizes for all treatment groups across the experiment. Note that no bioluminescence signal was present in the paromomycin or the WR279396 treatment group on day 8 after the end of treatment, even though the lesions had not yet healed. Detection of a bioluminescence signal on days 67 and 118 after the end of treatment indicates a relapse for two mice belonging to the paromomycin-treated group.

We utilized the *in vivo* imaging technology to assess the efficacy of a topical antileishmanial investigational cream (WR279396, which is a combination of paromomycin and gentamicin) and a cream with paromomycin only, which is currently being tested in humans under investigational new drug application IND 50098. Both topical creams and a vehicle control (consisting of the cream excipients without the active pharmaceutical ingredients) were applied twice daily (BID) for 10 consecutive days to BALB/c mice with established L. major lesions. Both WR279396 and the paromomycin-only cream were shown to significantly reduce the L. major parasite load below the level of detection by the 10th and last day of treatment, and the lesions were completely healed 10 to 12 days after the end of treatment. On day 8 after the end of treatment, two animals in the vehicle-control group showed a measurable parasite load in the ear (where the ear tag was inserted) and in the popliteal lymph node, respectively (Fig. 6). Shortly after that, all BALB/c mice belonging to the vehicle-control group were euthanized because of big lesion sizes. The bioluminescence signals remained below the limit of detection for all animals in the WR279396 and paromomycin-only cream treatment groups until day 67 after the end of treatment, when a BALB/c mouse belonging to the paromomycin-treated group relapsed and parasites were visible at the edge of the scar on the site of the original lesion. Two weeks later, this animal had lost 18% of its body weight and was euthanized because it was dehydrated, lethargic, and moribund. The bioluminescence signal continued to remain below the limit of detection for the remaining animals until day 118 after the end of treatment, when disease recurred at the same location in another BALB/c mouse belonging to the paromomycin treatment group (Fig. 6). All animals were euthanized on day 120 after the end of treatment.

### Assessment of treatment efficacy of some known antileishmanials in models of lesion cure in transgenic L. major BALB/c mice and GSH.

BALB/c mice and GSH were infected at the base of the tail with luciferase-expressing L. major parasites as described in the Materials and Methods section. All treatments were given i.p. for 10 consecutive days, except for paromomycin, which was given for i.p. for 7 days, and the two topical creams, the paromomycin and WR279396 creams, which were applied topically BID.

In BALB/c mice, lesions were allowed to grow for approximately 5 to 6 weeks until the mean lesion size reached an average of 50 mm^2^. Antileishmanial drugs used to treat the disease in humans, such as AmBisome, ABLC, amphotericin B, and the topical paromomycin and WR279396 creams, were tested and showed efficacy in this screen by healing or reducing established lesions in BALB/c mice in a dose-dependent manner. Twenty-five and 12.5 mg/kg AmBisome resolved established lesions in 5/5 and 4/5 BALB/c mice, respectively. Amphotericin B was tested at 16, 8, and 6 mg/kg and at these doses healed the lesions in 5/5, 3/5, and 1/5 mice, respectively, while 37.5, 25, and 12.5 mg/kg ABLC healed established lesions in 5/5, 5/5, and 4/5 mice, respectively. Both topical creams cured the lesions in 5/5 BALB/c mice (Table 2).

**TABLE 2:** Lesion cure in BALB/c mice and GSH treated with known antileishmanial compounds^*a*^

Compound tested	Dose (mg/kg)	No. of cured animals/total no. of animals tested	
		BALB/c mice	GSH
AmBisome	12.5	4/5*	5/5*
AmBisome	25	5/5*	5/5*
Amphotericin B	6	1/5	2/6
Amphotericin B	8	3/5*	5/6*
Amphotericin B	16	5/5*	6/6*
ABLC	12.5	4/5*	NT
ABLC	25	5/5*	NT
ABLC	37.5	5/5*	NT
Paromomycin	50	NT	3/5*
Topical paromomycin		5/5*	NT
Topical WR279396		5/5*	5/5*

a Five mice and five GSH were assigned to each treatment and vehicle-control group, except for the amphotericin B study, in which six GSH were assigned to each group. Treatment was initiated when lesions progressed to an average size of 50 mm^2^ and 150 mm^2^ for BALB/c mice and GSH, respectively. Drugs were given i.p. for 10 consecutive days, except for paromomycin, which was given i.p. for 7 days, and the two topical creams, paromomycin and WR279396, which were applied topically BID. The starting mean lesion sizes in the treatment groups were not statistically significantly different. Single-factor ANOVA (Dunnett's test) was used to determine whether the differences in the mean group lesion size existed on days 10 and 25 after the end of treatment for BALB/c mice and GSH, respectively. *, statistically significant difference (*P* < 0.05). Of note, all cures in GSH treated with known antileishmanial compounds happened before any of the BALB/c mice belonging to the vehicle-control group had self-healed. NT, not tested.

**FIG 7:**
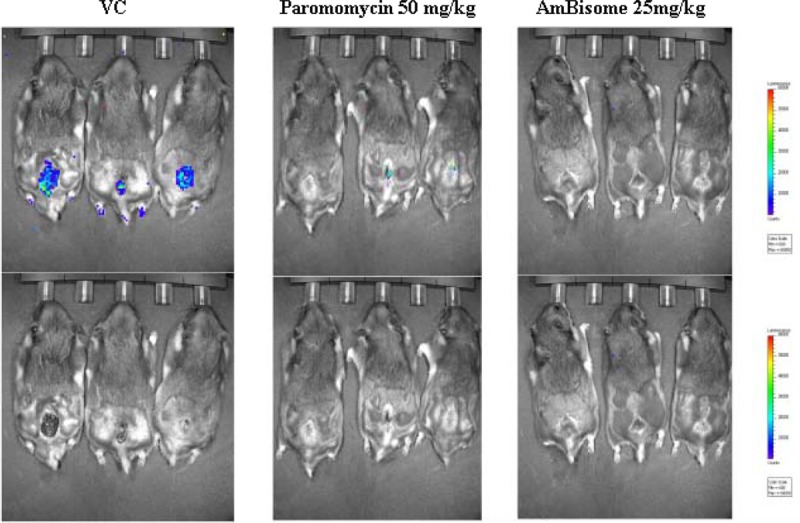
Efficacy of paromomycin (50 mg/kg) and AmBisome (25 mg/kg) in a second-tier Golden Syrian hamster model of lesion cure. Five GSH were assigned to each treatment and vehicle-control group. GSH were infected i.d. with 2 × 10^7^ stationary-phase luciferase-expressing L. major parasites at the base of the tail, and lesions were followed until the mean lesion size was approximately 150 mm^2^, at which time the animals were assigned to three different study groups. Paromomycin was given at 50 mg/kg and AmBisome was given at 25 mg/kg i.p. for 7 and 10 consecutive days, respectively. A single-factor ANOVA (Dunnett's test) was used to determine whether differences in group means existed across the experiment. Photon intensity measurements in the brighter, red areas of the image are larger than those in the dimmer, green-blue areas. A bioluminescence signal was detected at the infection site in all groups starting from day 0 postinfection and continued to be present in the vehicle-control group until the end of the study. A lack of a bioluminescence signal at the infection site suggested that the parasite load was nonexistent or below the limit of detection. The bioluminescence signals and lesion images shown here were taken on day 25 after the end of treatment. The same three animals are shown in the images at the top and bottom. Only the results for three GSH in each treatment group are shown.

Immunocompetent GSH were susceptible to the L. major infection at the base of the tail. Infections with luciferase-expressing L. major parasites routinely yielded a 100% infection rate. At 7 to 8 weeks postinfection, the mean lesion sizes reached approximately 150 mm^2^, at which time drug treatments were initiated. Twenty-five and 12.5 mg/kg AmBisome resolved all established lesions in GSH by 18 and 31 days, respectively, after the end of treatment, well before the GSH belonging to the vehicle-control group started self-healing. Treatment with 16, 8, and 6 mg/kg amphotericin B resulted in complete healing of 6/6, 5/6, and 2/6 hamsters, respectively, by day 34 after the end of treatment; none of the GSH belonging to the vehicle-control group had self-healed at that time. Paromomycin at 50 mg/kg resolved 3/5 lesions in GSH at 31 days after the start of treatment, for an overall reduction in the mean lesion size of 75%. No GSH belonging to the vehicle-control group demonstrated self-healing of lesions at that time. Topical WR279396 was observed to be effective in healing all established lesions in the GSH model of lesion cure by day 22 after the end of treatment; no self-healing was observed at this time in GSH belonging to the vehicle-control group (Table 2; Fig. 7). Some of the GSH belonging to the vehicle-control group self-healed before day 60 after the end of treatment, at which point all animals were euthanized. For both animal models of lesion cure, we chose to use AmBisome as a positive control at a dose of 25 mg/kg.

## DISCUSSION

We previously published a multitiered drug screening paradigm that suggested testing of compounds with strong *in vitro* antileishmanial potency and good chemical characteristics (low or no *in vitro* toxicity, favorable stability in liver microsomes, acceptable permeability, etc.) in a first-tier lesion suppression efficacy model in highly susceptible immunodeficient BALB/c mice (28). Efficacious compounds can then be tested further in more traditional lesion cure models in mice and hamsters that have CL with clinical similarities to the human form of CL (28). In order to provide a higher-throughput BALB/c mouse model of lesion suppression, we explored the use of optical imaging to establish an animal model of the efficacy of drugs against CL with the shortest possible cycle time to enhance our testing throughput.

It has been established that there is a significant correlation between parasite load and lesion size in BALB/c mice infected with Leishmania parasites (29, 30). Our studies take these findings further and show that there is also a strong correlation between the parasite load and luciferase activity, measured using an *in vivo* imaging technology, during the first 3 weeks postinfection in BALB/c mice infected at the base of the tail with L. major parasites. More importantly, our study confirms that a significant reduction in the bioluminescence signal in the early stages of infection strongly correlates with the development of a significantly smaller lesion in advanced-stage CL. In our study, most known antileishmanial drugs were found to have strong *in vitro* and *in vivo* antileishmanial potencies against intracellular amastigotes and in the animal models, respectively. Paromomycin, sinefungin, posaconazole, and all three formulations of amphotericin B showed excellent efficacy in the new BALB/c mouse model of lesion suppression, with AmBisome being the most efficacious. The chemotherapeutic efficacy of glucantime, sodium stibogluconate, miltefosine, and pentamidine against Leishmania spp. appears to be at least partially dependent upon the development of an effective immune response (1, 31–34). This might be one of the reasons for the total or partial lack of efficacy of these drugs in BALB/c mice, which lack an early NK cell and a Th1-type response (35–38). As mentioned above, pentamidine injected i.p. showed no efficacy when it was tested in this model. Interestingly, changing the route of administration from i.p. to i.m. significantly increased the efficacy of this drug. Treatment with miltefosine, glucantime, and fluconazole, which have poor *in vitro* activities against the L. major strain tested, appeared to be ineffective in our lesion suppression model (1). It is possible that longer-term treatment with miltefosine or fluconazole, which have clinical efficacy in CL patients when given at high doses for a long duration, might still be effective in the context of a longer treatment in BALB/c mice (39, 40). Clearly, successful *in vivo* testing of compounds requires information about the particular class of compound being tested to include information regarding drug pharmacokinetics, a reasonable idea about the length of treatment, the route of administration, and potential differences in efficacy toward Leishmania spp. The implementation of the *in vivo* imaging technology in the lesion suppression model described here significantly decreased the length of time of our initial testing cycle, which makes this testing method rapid and cost-effective. From an animal welfare perspective, a more rapid and efficacious assay keeps animal pain and suffering to a minimum.

Several known antileishmanial drugs and topical creams were used to validate the second-tier BALB/c mice and GSH models of lesion cure. AmBisome, ABLC, and amphotericin B healed and reduced the size of established lesions of L. major in a dose-dependent manner. Topical paromomycin and WR279396 were efficacious and healed established lesions in mice and GSH. Even though bioluminescence signal measurements cannot be used as an experimental endpoint in the lesion cure models, our study suggests that the *in vivo* imaging technology can be used to follow the evolution of the parasite load and assess drug efficacy in advanced stages of CL in animals that have established lesions. Furthermore, by being able to visualize the presence or absence of parasites at the site of a previously healed lesion, we can determine a possible relapse several weeks before the clinical signs of disease reappear. Promising lead compounds derived from the *in vivo* screens described in this study can be further tested in a third- and last-tier model of lesion cure in BALB/c mice and GSH infected with transgenic New World Leishmania parasites. These models are in the last stages of being validated in our laboratory. We believe that the array of *in vivo* animal models that we have developed, using both Old World and New World Leishmania spp., provides a complete set of tools to evaluate compounds for anti-CL drug discovery (28).

## MATERIALS AND METHODS

### Parasite culturing and animal infection.

L. major promastigotes (NIH173 [MHOM/IR/−/173]) were harvested from infected BALB/c mouse footpads and were cultured in Schneider's medium (Lonza Life Sciences, Walkersville, MD) supplemented with 20% heat-inactivated fetal bovine serum. Cultures were maintained in T75 tissue culture flasks (Corning Life Sciences, Manassas, VA) at 22°C. Promastigotes for infection were harvested from the culture by spinning it at 2,000 rpm for 20 min. The medium was removed, and the resulting pellet was suspended in 1× PBS. Two additional spins at 2,000 rpm were conducted in PBS. After the second spin, a low volume of PBS was added, and stationary-phase promastigotes were counted and suspended at 1 × 10^8^ parasites/ml. Animals were infected at the base of the tail with 100 and 200 μl of parasite culture containing 1 × 10^7^ and 2 × 10^7^ L. major luciferase-expressing stationary-phase promastigotes, respectively, for BALB/c mice and GSH, respectively.

### Animals.

Female BALB/c mice weighing 20 to 25 g and male GSH weighing 50 to 70 g were purchased from Charles River Laboratories (Wilmington, MA). All animals were purchased from the same vendor to prevent possible genetic variations that may occur by the use of animals from a different colony, which may interfere with the results.

### Ethics statement.

The animal protocol for this study was approved by the Walter Reed Army (WRAIR) Institute of Research Institutional Animal Care and Use Committee (protocol number 13-ET-26) in accordance with national and U.S. Department of Defense (DoD) guidelines. The research was conducted in an AAALACi-accredited facility in compliance with the Animal Welfare Act and other federal statutes and regulations relating to animals and experiments involving animals and adheres to principles stated in the *Guide for the Care and Use of Laboratory Animals* (41).

### Drugs.

Drugs, including a topical treatment comprised of two aminoglycoside antibiotics (paromomycin and gentamicin [WR279396]), a paromomycin cream, and a vehicle-control cream, were ordered from the Division of Experimental Therapeutics, WRAIR chemical library, Rockville, MD. Compounds not found in the WRAIR chemical collections were acquired commercially. AmBisome was purchased from Astellas Pharma, US Inc. (Northbrook, IL). Amphotericin B lipid complex (ABLC) was obtained from Enzon Pharmaceuticals, Inc. (Piscataway, NJ). Amphotericin B for injection USP was purchased from X-Gen Pharmaceuticals, Inc. (Horseheads, NY).

### Generation of the bioluminescent L. major parasite.

L. major NIH173 (MHOM/IR/−/173) was a gift from Genevieve Milon, Department of Parasitology and Mycology, Institute Pasteur, Paris, France (14, 18).

### *In vitro* characterization of luciferase-expressing L. major parasites.

To assess the *in vitro* correlation between the number of luciferase-expressing L. major parasites and luciferase activity, parasite cultures were concentrated to 1.18 × 10^9^ parasites/ml in 1× PBS. Two hundred microliters of parasite culture was added to the first well of a Nunc 96-well polystyrene white flat-bottom plate, and parasites were 2-fold serially diluted across the plate in 1× PBS. The parasite concentrations within the plate ranged from 1.18 × 10^8^ to 140 parasites per well. Ten microliters of luciferin solution previously diluted to 150 μg/ml was added to each well, and the plates were incubated for 30 min in the dark. Luminescence values were determined using a Tecan Infinite M200 plate reader (Tecan Inc., Durham, NC). The *in vitro* potencies (IC_50_s) of known antileishmanial compounds were determined using an intracellular amastigote assay that was developed in our laboratory and described elsewhere (26). This well-characterized assay utilizes luciferase activity as a measure of L. major parasite proliferation (26). The *in vitro* cytotoxicities (IC_50_s) of known antileishmanial compounds against the murine macrophage cell line RAW 264.7 (ATCC TIB-71) were tested using a 3-(4,5-dimethylthiazol-2-yl)-2,5-diphenyltetrazolium bromide (MTT) cytotoxicity assay as described in reference 42. RAW 264.7 cells for this assay were cultured in Dulbecco modified Eagle medium (catalog number 30-2002; ATCC) supplemented with 10% heat-inactivated fetal bovine serum (catalog number 16140-089; Invitrogen). The IC_50_ determinations were generated for each dose-response test using GraphPad Prism software (GraphPad Software Inc., San Diego, CA) using the nonlinear regression (sigmoidal dose-response/variable slope) equation.

### *In vivo* parasite load measurements.

The parasite load was measured as described in reference 43. BALB/c mice were infected intradermally (i.d.) at the base of the tail with 1 × 10^7^ stationary-phase luciferase-expressing L. major parasites. Three animals were randomly chosen and euthanized on days 1, 3, 6, 9, 14, and 18 postinfection. Before sacrifice, the bioluminescence signal was measured using a PerkinElmer IVIS Spectrum instrument set at the medium-binning and automatic exposure settings (see “*In vivo* bioluminescence imaging of luciferase-expressing L. major parasites” below). Immediately after euthanasia the affected tissue was excised from the dorsolumbar region using a punch biopsy. The biopsy specimens were weighed and then ground in 10 ml Schneider's medium (Lonza Life Sciences, Walkersville, MD) supplemented with 20% heat-inactivated fetal bovine serum using a ProScientific 300D homogenizer. Tissue homogenates were centrifuged for 5 min at 500 rpm, the supernatant was serially diluted in Schneider's medium (dilution factor, 4), and the serial dilutions were cultured in 96-well plates. The plates were incubated for 5 days, and each well was then examined and classified as positive or negative according to whether or not viable promastigotes were present.

### *In vivo* bioluminescence imaging of luciferase-expressing L. major parasites.

Luciferin (d-luciferin potassium salt; Xenogen Corporation, Alameda, CA, and Gold Biotechnology, St. Louis, MO), the luciferase substrate, was inoculated intraperitoneally (i.p.) into BALB/c mice and GSH at a concentration of 200 mg/kg at 18 min before bioluminescence analysis. Animals were anesthetized in a 2.5% isoflurane atmosphere (MWI Veterinary Supply, Harrisburg, PA) for 7 min and maintained in the imaging chamber for analysis. Emitted photons were collected by automatic acquisition with a charge-coupled-device (CCD) camera (PerkinElmer IVIS Spectrum *in vivo* imaging system) using the medium-resolution (medium-binning) mode. Analysis was performed after a region of interest (ROI) that delimited the surface of the affected area was defined. The total photon emission from the infected area at the base of the tail was quantified with Living Image software (Xenogen Corporation, Alameda, CA), and results were expressed in numbers of photons per second.

### Establishment of a first-tier Leishmania lesion suppression screening model using *in vivo* imaging in BALB/c mice infected with luciferase-expressing L. major parasites.

On day 0, BALB/c mice were infected i.d. at the base of the tail with 1 × 10^7^ L. major luciferase-expressing stationary-phase promastigotes as described before (see “Parasite culturing and animal infection” above). Five mice were assigned to each treatment, positive-control, and vehicle-control group. At 3 days postinfection and immediately prior to treatment, the baseline parasite load was determined by measuring the bioluminescence signal. The compounds were administered i.p. to all groups, including the positive-control and the negative-control groups, except for pentamidine, which was administered both i.p. and i.m. Drug dosages were chosen on the basis of the human-equivalent dose (HED), and dosing calculations were based upon body weight measurements obtained on day 3 postinfection. Cold (4°C) double-distilled sterile water (ddH_2_O) was used to prepare fresh stocks of AmBisome, ABLC, and amphotericin B. All other compound solutions were prepared fresh before drug administration by dissolving the needed quantity of drug in cold (4°C) 0.5% (wt/vol) hydroxyethyl cellulose and 0.2% (vol/vol) Tween 80 (0.5% HECT). If needed, drugs were ground using a ProScientific 300D homogenizer, and the particle size was measured using a Horiba LA-950V2 particle size analyzer. Treatments usually lasted 10 days. In order to accommodate treatments that might be longer or shorter (7 to 14 days), for all the experimental groups bioluminescence was measured on day 18 postinfection, and the mean number of photons per second was calculated. Results were expressed as percent suppression of the bioluminescence signal compared with that for the vehicle-control group.

### Establishment of a second-tier Leishmania lesion cure screening model in BALB/c mice and immunocompetent GSH infected with luciferase-expressing L. major parasites.

Some of the promising antileishmanial compounds, defined by a bioluminescence signal suppression assay result showing a ≥50% reduction in the parasite load in the primary *in vivo* screen, were further tested in two consecutive second-tier lesion cure tests in BALB/c mice and GSH. BALB/c mice and GSH were infected i.d. at the base of the tail with 1 × 10^7^ and 2 × 10^7^ L. major luciferase-expressing stationary phase promastigotes, respectively. Starting from the second week postinfection, the lesion induration diameters (where D1 is the length and D2 is the width) were measured using a caliper instrument (Fisher Scientific, USA) with a 0.1-mm sensitivity. Length and width measurements were taken to account for asymmetrical lesions. The lesion size area was then calculated using the formula πR1 · R2 (where R1 is equal to D1/2 and R2 is equal to D2/2). Treatment was initiated when the lesions progressed to average sizes of 50 mm^2^ and 150 mm^2^ for BALB/c mice and GSH, respectively. Except for topical paromomycin, WR279396, and their vehicle controls, which were applied topically BID, all other drugs were given i.p. The treatments lasted 10 days, except for the treatment with paromomycin, which was given for 7 days. Dosing calculations for drugs given i.p. were based upon body weight measurements obtained on the first day of treatment. Drugs were prepared as described above in “Establishment of a first-tier Leishmania lesion suppression screening model using *in vivo* imaging in BALB/c mice infected with luciferase-expressing L. major parasites.” Results were expressed as percent suppression of the mean lesion size compared with that for the vehicle-control group. One hundred percent suppression (lesion cure) was considered 100% reepithelialization of the lesion (lesion size, 0 by 0 mm^2^).

### Use of an optical imaging technology in determining the efficacy of topical antileishmanial drugs in the BALB/c mouse model of lesion cure.

An *in vivo* optical imaging technology was also used to assess the efficacies of compounds applied by the topical route. Three creams were tested for efficacy and safety in well-established animal lesions. BALB/c mice were infected i.d. at the base of the tail with 1 × 10^7^ stationary-phase luciferase-expressing L. major parasites. The lesions were allowed to progress until they reached an average size of 50 mm^2^ and were debrided the day before the treatments started. Five mice per treatment were randomly assigned to groups in which the mean lesion sizes were not statistically significantly different. Before each topical treatment, residual gels from the previous treatments were removed using clean cotton-tipped applicators, and the lesions were thoroughly cleaned with a 1× PBS-soaked gauze. Sterile gauze sponges were used to dry the lesions after they were washed with 1× PBS. Topical creams were applied twice daily for 10 consecutive days. Lesions were examined and measured at intervals of 7 ± 2 days. Lesion suppression results were expressed as a percentage of the mean lesion size compared with that for the vehicle-control group. Lesion cure was considered 100% reepithelialization of the lesion or a lesion size of 0 by 0 mm^2^. A reduction in the parasite load or a lack thereof was also followed by measuring the bioluminescence signal for all treatment groups.

### Statistics.

In the lesion suppression screen, five mice were randomly assigned to each treatment and control group. For the L. major lesion cure screens, five BALB/c mice or five to six GSH were assigned to groups in which the average lesion sizes were not statistically significantly different. One-way analysis of variance (ANOVA; Dunnett's test), which compares a number of treatment groups with a single control group (many-to-one comparison), was used to determine statistical significance and provide confidence intervals to detect differences between the mean for a number of treatments with the mean for the control group. Drug efficacy in the lesion suppression and lesion cure screens was evaluated by calculating the bioluminescence signal suppression and lesion size reduction in the treated groups compared to those for the vehicle-control (1× PBS)-treated group, respectively. A *P* value of <0.05 was accepted for the purpose of declaring that the treatments had statistically significant effects.

## Supplementary Material

Supplemental material
