# Supplementary material for: Use of Optical Imaging Technology in the Validation of a New, Rapid, Cost-Effective Drug Screen as Part of a Tiered In Vivo Screening Paradigm for Development of Drugs To Treat Cutaneous Leishmaniasis
Source: Antimicrob Agents Chemother. 2017 Mar 24;61(4):e02048-16. doi: 10.1128/AAC.02048-16 (PMC5365718; doi:10.1128/AAC.02048-16)
Supplement: Supplemental material [file supp_61_4_e02048-16__index.html]

Supplemental material 

# Use of Optical Imaging Technology in the Validation of a New, Rapid, Cost-Effective Drug Screen as Part of a Tiered *In Vivo* Screening Paradigm for Development of Drugs To Treat Cutaneous Leishmaniasis

## Supplemental material

- Supplemental file 1 -

  Supplemental Figure S1 and Table S1

  PDF, 181K
