# Supplementary material for: Use of Optical Imaging Technology in the Validation of a New, Rapid, Cost-Effective Drug Screen as Part of a Tiered In Vivo Screening Paradigm for Development of Drugs To Treat Cutaneous Leishmaniasis
Source: Antimicrob Agents Chemother. 2017 Mar 24;61(4):e02048-16. doi: 10.1128/AAC.02048-16 (PMC5365718; doi:10.1128/AAC.02048-16)
Supplement: Supplemental material [file AAC.02048-16_zac004176048s1.pdf]

**Figure S1: *In vitro* correlation between the log- transformed values of relative luminescence units (RLU) and log transformed values of the number of luciferase-expressing *L. major* parasites.** Two hundred µL of parasite culture was added to the first well of a Nunc 96-well polystyrene white flat bottom plate, and parasites were two-fold serial diluted across the plate in 1X PBS. Parasite concentrations shown here ranged from 1.18x10^8 to 140 parasites per well. Ten µL luciferin solution previously diluted to 150µg/mL was added to each well and plates were incubated for 30 minutes in the dark**.** Emitted luminescence signal is measured using a Tecan Infinite M200 plate reader and expressed as RLU.

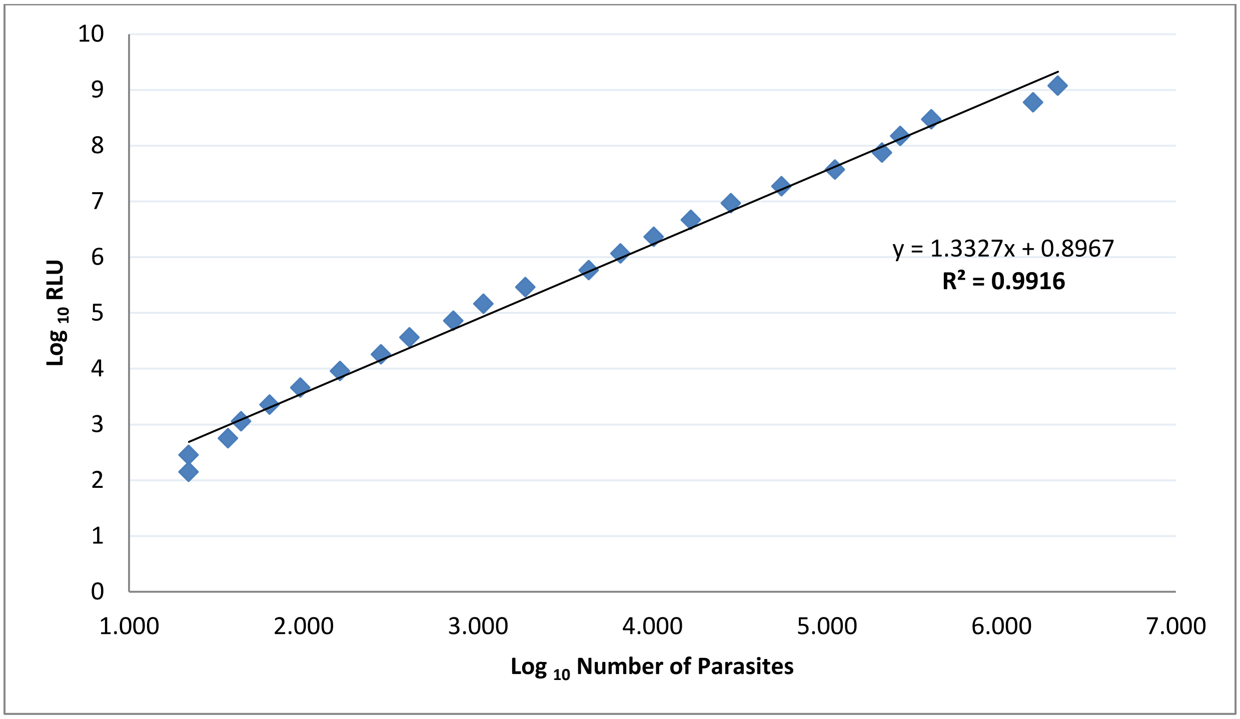

**Table S1: The *in vitro* potency and cytotoxicity inhibitory concentrations ($IC_{50}$) of known anti-leishmanial compounds*.**

| Compound | *In vitro* Potency $IC_{50}$ (ng/ml) | *In vitro* Toxicity $IC_{50}$ (ng/ml) |
|---|---|---|
| Pentostam | >2000 | >15151 |
| Paromomycin | 554.3±222.6 | >15151 |
| Amphotericin B | 25.3±11.6 | >15151 |
| Pentamidine | ND | 1622.5±86.54 |
| AmBisome | 29.7±15.1 | >15151 |
| Glucantime | >10000 | >15151 |
| Fluconazole | >10000 | >15151 |
| Ofloxacine | >10000 | >15151 |
| Posaconazole | 147.4 | 1483.3±141.2 |
| Miltefosine | 222.9±10.8 | >7575 |
| Sitamaquine | 4164.9±154.1 | >15151 |
| Sinefungin | NT | >15151 |

* In the intracellular amastigote assay all drugs were tested in triplicates except for Posaconazole that was tested as a single $IC_{50.}$ In the *in vitro* cytotoxicity assay all drugs were tested in duplicates. [&] The i*n vitro* potency for Pentamidine could not be determined because of the high toxicity of this compound in the RAW macrophage cell line. ND=Not determined. NT=Not tested.
